# The current state of Carceral health data: an analysis of “Listening Sessions” with stakeholders

**DOI:** 10.1186/s40352-023-00239-4

**Published:** 2023-09-28

**Authors:** Zaire Cullins, Michael Forrest Behne, Lauren Brinkley-Rubinstein

**Affiliations:** School of Medicine, Department of Population Health Sciences, Duke University, Durham, USA

**Keywords:** Data availability, Carceral health, Incarceration

## Abstract

**Background:**

Understanding the health conditions of those under carceral control is often made difficult due to lack of access to data. Yet, as has been made clear during the COVID-19 pandemic, is that data is essential to understand the scope of disease and how best to allocate resources. To better understand the needs of criminal legal oriented research and non-profit organizations, we interviewed stakeholders to better understand how they use existing data, what data they lack, and what data they would like to have to optimally assess the health of people who are incarcerated.

**Results:**

Stakeholders reported a lack of trust and data availability as key issues. Many perceived the few institutions that do collect and disseminate data as obfuscating data or having a bias in collection and reporting. Additionally, concerns such as balancing the interest of systems-impacted people with advocacy were described as concerning for participants.

**Conclusions:**

To tackle these issues of transparency and availability, the authors believe that an independent oversight body could be instrumental to ensuring accurate and timely data collection and reporting. As many participants turned to creating their own data, coalition building could be influential as a large network of resources may support capturing the varied experiences of people who are incarcerated.

## Background

The United States has the world’s highest incarceration rate and maintains the largest number of people involved in the criminal legal system (Nowotny et al., 2021). Though we do not have an exact number of individuals incarcerated in jails and prisons at any one moment, an estimated 6.7 million individuals in the United States are under direct carceral control (Nowotny et al., 2021) For most, non-incarcerated sub-populations in the United States, some understanding of individual and community health can be attained (CDC, n.d.). Researchers might use large, nationally representative surveys or administrative data from electronic medical records, yet for carceral populations, investigating health conditions on a large-scale is nearly impossible. These same kind of large datasets largely do not exist.

With major health crises emerging such as COVID-19 and monkeypox, the lack of data transparency within carceral facilities has been spotlighted (Barnert et al., 2021; Brinkley-Rubinstein et al., 2022). Any understanding of carceral health is currently achieved primarily through three data domains: national datasets, administrative data, and public records. The Bureau of Justice Statistics (BJS), under the auspices of the Department of Justice, administers national surveys to carceral agencies (Kluckow & Zeng, 2022). However, findings are reported at a years-long lag, precluding any real-time insights by observers (Peterson & Brinkley-Rubinstein, 2021). The BJS report, “Correctional Populations in the United States, 2017–2018,“ was only released in August of 2020 and data from 2019 was released in 2021 (Kluckow & Zeng, 2022; Minton et al., 2021).

Administrative data derived from state carceral agencies poses additional, distinct challenges for analysis by researchers. Certain Departments of Correction (DOCs), predominately from ideologically progressive states such as California and Rhode Island, proactively share information pertaining to the health of their confined populations (*Reports & Court Orders*, 2023; Dumont et al., 2021). Other states–including Arkansas, Mississippi, and Louisiana–remain opaque to outside observers, thus creating bias in available data (Loyola Law Professor Releases Report on State of Healthcare Services in Louisiana Prisons | Loyola University New Orleans, 2021). Some researchers have utilized public records requests in attempts to glean health data from carceral agencies (Behne et al., 2022). These efforts make use of Freedom of Information Act (FOIA) to solicit documents from the federal government as well as similar state-level records requests (Behne et al., 2022). While obtaining certain types of information successfully, this strategy may be costly and inefficient with requests frequently taking years before receiving responsive documents (GovQA, 2021).

The enduring legacy of the criminal-legal system’s origin has led to disparate carceral encounters on the basis of race, ethnicity, national origin, gender, and sexuality (Brinkley-Rubinstein & Cloud, 2020; Baćak et al., 2018). Members of these minoritized communities are disproportionately represented in arrests and incarceration (Dumont et al., 2013). Available and high-quality data on the health outcomes for those who come in contact with systems of punishment are therefore important to improving health equity, as incarceration may worsen pre-existing conditions that these populations already experience due to issues such as poverty outside of confinement. (Brinkley-Rubinstein, 2013).

Given these issues with data availability and accuracy combined with the urgent need to understand the impact of carceral systems on health, we spoke with organizations in the carceral health space about data. During interviews, we sought to elucidate the existing resources for data, the use and understanding of carceral health data, and explore the organizations’ needs for health data.

## Methods

We conducted 22 hour-long interviews with stakeholders who worked for organizations dedicated to criminal legal advocacy or individuals in the academy whose research interests pertain to criminal-legal involvement. These semi-structured interviews were designed as listening sessions, wherein prompts were intentionally open-ended and allowed the interviewee to freely share their thoughts. Organizations selected for recruitment were based in the United States and were involved in research, advocacy (direct or legal), community support, and/or journalism pertaining to individuals or groups with involvement in the criminal-legal system (n = 90). Recruitment was further restricted by organizations with an online presence and easily-retrievable contact information for email outreach (n = 52).

Recruitment for the listening sessions occurred via email outreach using a standardized template approved by the University of North Carolina at Chapel Hill institutional review board. Organizations which met eligibility criteria (n = 52) were contacted by email in accordance with IRB procedures. A 6-month recruitment window was selected to reflect contemporaneous experiences with both the pandemic and changes to the criminal-legal landscape. All eligible participants who were able to schedule a listening session within the 6-month recruitment window were accepted into the study.

All participants provided informed consent electronically and gave verbal consent to be recorded electronically during the interview conducted over Zoom. The subsequent audio file was submitted to Rev.com for anonymized transcription, a condition of participation in the study. Of the 22 participants recruited, 36.3% identified as representatives of advocacy organizations (n = 8), 40.9% identified as researchers (n = 9), 9.09% worked for community-support organizations (n = 2), 9.09% were members of the legal community (n = 2), and 4.55% worked for journalistic outlets (n = 1). Participants were based in 11 states from the Northeast, Southeast, Southwest, and Midwest regions of the United States. Participants were not compensated for their time. Recruitment and interviewing occurred on a rolling basis between January 2021 and July 2021.

Many organizations that were interviewed narrowed their foci to specific interests within the incarcerated population, such as mental health, advocacy, data transparency, and reform. Most of the organizations operated on a national or state-level with a few working in specific regions or internationally. Most stakeholders interviewed were affiliated with a community organization rather than an academic institution, with most engaged in either research or advocacy work.

A general inductive framework was used to create preliminary codes from the interviews, and the final codebook was composed utilizing open and axial coding. Two researchers then independently coded each anonymized transcript, applying the codebook in the web-based qualitative and mixed methods software Dedoose. Outstanding discrepancies between coded transcripts were resolved by a third researcher.

## Results

Following the coding of all transcripts, several major themes emerged. Many participants reported that they felt a lack of trust in the institutions that collect and disseminate data, perceiving a fundamental bias or unwillingness to commit to transparency. Participants also reported encountering ethical tensions between performing research and advocating for marginalized populations. Such concerns included the difficulty balancing the interests of systems-impacted people with the activities advocates felt were required to obtain usable data. Relatedly, many participants perceived a lack of data availability across the criminal-legal landscape. Consequently, the few available and reliable datasets were resources shared by nearly all groups that engaged in the interviews—a fact revealed by the present study. Each of these is discussed in more detail below.

### Distrust in institutions

Nearly all (n = 20) participants utilized data provided by government institutions in the course of their work, A majority (n = 16) of stakeholders, however, expressed a present distrust in the government data or reported that they had previously encountered inaccurate data reported from government sources. Some reasons that stakeholders may not trust data reported by government entities or the carceral facilities themselves include the closed off nature of carceral facilities, making it so that oversight of data collection practices is very difficult, the lag between when data are published by the government compared to when the data were collected, and the disproportionate use of carceral systems against historically disadvantaged populations. Due to this distrust, there were several organizations that combined their data from the government with other data sources such as publicly available contracts or news articles. Several participants expressed beliefs that the data were “subject to manipulation” stating that “jails will send people to the hospital at the last minute and they’ll die there and then claim it wasn’t in-custody death.” One participant stated that, at the beginning of the COVID-19 pandemic, one New Jersey jail “had a significant ICE detainee population…just took [the ICE detainees] off the roster,” allegedly in response to a “decarceration effort in March [of 2020].” Situations such as these have led to an uneasiness between our participants and the government and carceral facilities data that they use.

### Ethical issues

Participants also reported a variety of ethical tensions with their work. The most reported ethical concerns were issues related to data authenticity (n = 11) and the tension of balancing their research efforts with their advocacy work (n = 7). When it comes to the authenticity of data, participants were skeptical about how these institutions were reporting. One explained that they think “data is always, always, always skewed and biased by what police want to report versus what they don’t want to report, what prisons want to share versus what they don’t”. Therefore, participants feel stuck as they have no data to rely on, besides the government or carceral facilities data that they already do not trust. But, without any data, the participants may face issues of credibility when speaking about the conditions and practices inside of carceral facilities.

Additionally, many participants reported that they felt hesitant to collaborate with the government entities that operate carceral facilities, especially as many of the interviewees reported having an abolition outlook and maintain that ending systems of incarceration is the goal of their efforts. Using data from carceral facilities while arguing for the abolition of these facilities poses a unique tension for the abolition-focused participants as they collaborate with institutions that have caused harm in the population they have likely built relationships with. When arguing for the defunding and abolition of carceral facilities, while also pointing out that these facilities don’t publish data, one risks these institutions receiving more funds to publish more data, which could enable them to cause even more harm. One participant added “It’s how far do you go to get access to data, to get access to these places”. Another participant articulated their complicated relationship with Department of Corrections by saying “you have to be very smart about the data sharing agreements that you write and make sure that it’s not going to stifle your ability to publish or stifle your ability to share what you learn”. As these participants have highlighted, when they begin developing relationships with Departments of Corrections, they must balance their integrity as researchers while potentially partnering with institutions they fundamentally disagree with.

Participants also reported feeling apprehensive about these relationships as many of them had worked diligently to cultivate a relationship with the population that they were studying. One participant mentioned that they found themselves “talking to communities and making sure that recommendations that I was making or advocating for were protective of them and did nothing to restrict their autonomy and their freedom.”, showing the balancing act that researchers in the field must perform – protecting the population that they seek to help while also collaborating with the harmers.

Many other ethical concerns were reported by the participants such as how to navigate the influx of attention organizations get after a tragedy reaches the national news cycle, making sure that they can publish the data they obtain while respecting the dignity of those involved, and including the populations that they study within their work. One participant reflects on the privacy issue, stating “there’s some people who might say that we shouldn’t be putting out names of individuals, but that’s part of the report”. One example where a participant recounts an uncomfortable situation they experienced, noting how “There’s a county…in Texas that posts full names and relations of all jail visitors. So there’ll be like…a daughter of somebody who’s incarcerated for whatever reason, now her name is just on a jail roster in perpetuity. Like that’s absurd to me.” Overall, participants reported several worries they had about how to best protect the people they are researching, wanting to avoid perpetuating even more harm to these communities.

### Lack of data availability

One of the most common complaints articulated by the participants was the lack of data. Nearly all 22 participants (n = 20) reported that the data they were interested in did not exist. One of the participants stated that they were “used to operating within a system of deprivation, where resources are of scarcity” Approximately three-quarters (n = 16) of interviewees reported the data they wanted were not publicly available. For one of the participants that focused on data in jails, they noted the difficulty of obtaining jail data stating that “there’s not a lot that [jails] publish, even if they collect,” thus hampering grassroots data aggregation efforts. The remaining participants did not address specific concerns with data availability.

In addition, a majority (n = 20) of participants reported using government sources for their data. Some of the most cited sources were reports from the Bureau of Justice Statistics, such as the Prisoner Statistics Program reports and the National Corrections Reporting Program reports, as well as reports issued by the Department of Corrections themselves. Participants whose organizational focus was more national in scope, lacked the interpersonal relationships with Department of Corrections stakeholders to gain access to non-public data.

## Discussion

When participants were asked about their wants and needs in carceral health data, many reported struggling with the same issues. Most agreed that there was a lack of data while also expressing distrust in the data that is currently available.

The COVID-19 pandemic demonstrated a capability for carceral agencies to collect and report health data on individuals in custody, exemplified by daily publications of public health metrics. Researchers hoped that the dashboards presenting this information would become a fixture of DOC websites, only for governments to stop reporting prison-specific coronavirus-related material entirely. Some reasons DOC websites may have stopped publishing data relate to a decline in public attention on the COVID-19 pandemic and/or their specific state ending the COVID-19 state of emergency. Since then, however, renewed calls for transparency and accountability have surged. In September of 2022, the Senate heard testimony on the failures of the Deaths in Custody Reporting Act to establish meaningful visibility into custodial mortality (*Uncounted Deaths in America’s Prisons and Jails: How the Department of Justice Failed to Implement the Death in Custody Reporting Act*, 2022). Members of the press have called for oversight into correctional practices after high-profile incidents affecting the health of confined individuals (Urell, 2022). Research and advocacy communities appear to recognize the current lack of available health data and are beginning to demand change.

Participants in this study consistently highlight the lack of available and reliable data on issues such as the health of people while they are incarcerated, budget information such as health expenditures, and operating procedures. Additionally, available data varies widely in variety of information due largely to jurisdictional regulations governing government transparency. Such idiosyncratic approaches are visible in the comparatively robust custodial mortality reporting systems is Texas whereas, in nearby Alabama, the DOC has stopped publishing monthly death data (*Custodial Death Report | Office of the Attorney General*, n.d.; (Davis, 2023). Therefore, stakeholders would benefit from some form of oversight beyond the institutions themselves that may encourage a more accurate and timely release of data. Consequently, multiple interviewees reported seeking out their own data, through means such as outreach in their local communities to make up for or supplement data collected at the institutional level. While the inefficiency of this process makes large-scale replication challenging for organizations with a regional or national focus, its investigatory value cannot be overstated. The results of these interviews suggest that the research and advocacy communities would substantially benefit from close collaboration with a broad network of shareholders. Smaller organizations with a local or regional focus are better equipped to foster and maintain relationships with those who can provide direct insight into carceral health, such as those formerly incarcerated. Partnerships between these organizations can further enhance our understanding when resources and results are shared. Through coalition building across varied and geographically dispersed organizations, the goals of uplifting experiential insights and enhancing the visibility of carceral health can be achieved.

## Conclusion

Health equity research in populations with criminal-legal involvement represents an area of urgency because this population faces a high degree of discrimination and historically marginalized groups are overrepresented. To better understand how to shape effective and responsive research and advocacy for issues impacting those involved in the criminal legal system, we conducted listening sessions with several organizations and individuals that touch multiple aspects of the criminal legal system or those most affected by it. Our analysis of these sessions showed that data accuracy and availability is one of the most important issues demonstrating a common perception of inadequate or missing data. Participants reported several ethical tensions when performing this work, such as concerns surrounding privacy and worries about inflicting further harm in the communities they seek to help. These interviews further the interests of the research community by enhancing study design and interventions that are most reflective of the needs of incarcerated populations and the advocacy community that serves them.

## Data Availability

The datasets used and/or analyzed during the current study are available from the corresponding author on reasonable request.
